# Supplementary material for: The Construction and Exploration of a Comprehensive MicroRNA Centered Regulatory Network in Foxtail Millet (Setaria italica L.)
Source: Front Plant Sci. 2022 May 6;13:848474. doi: 10.3389/fpls.2022.848474 (PMC9121102; doi:10.3389/fpls.2022.848474)
Supplement: Supplementary file 6 [file Data_Sheet_6.zip › Sit-miRN3001b_Seita.1G050700.1_2888_TPlot.pdf]

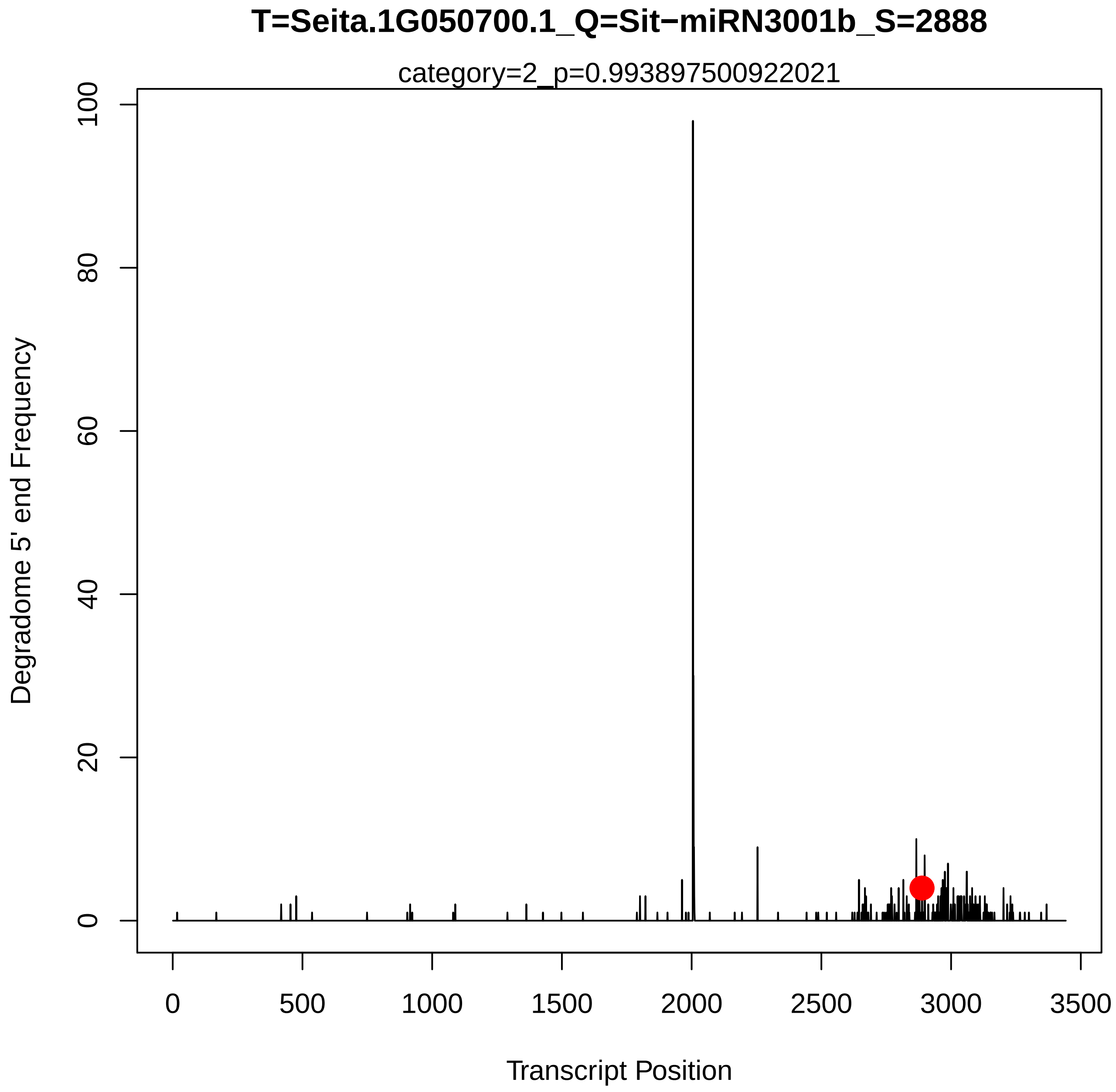
T=Seita.1G050700.1_Q=Sit−miRN3001b_S=2888
category=2_p=0.993897500922021
Degradome 5' end Frequency
0
20
40
60
80
100
0
500
1000
1500
2000
2500
3000
3500
Transcript Position
